# An open randomized clinical trial in comparing two artesunate-based combination treatments on *Plasmodium falciparum *malaria in Nigerian children: artesunate/sulphamethoxypyrazine/pyrimethamine (fixed dose over 24 hours) versus artesunate/amodiaquine (fixed dose over 48 hours)

**DOI:** 10.1186/1475-2875-9-378

**Published:** 2010-12-31

**Authors:** Idowu Adejumoke Ayede, Adegoke Gbadegesin Falade, Akintunde Sowunmi, Frans Herwig Jansen

**Affiliations:** 1 Department of Paediatrics, College of Medicine, University College Hospital, Ibadan, Nigeria; 2 Department of Pharmacology and Therapeutics, College of Medicine, University College Hospital, Ibadan; 3 Dafra Pharma, Turnhout, Belgium

## Abstract

**Background:**

Several studies have demonstrated the efficacy of artemisinin-combination therapy (ACT) across malaria zones of the world. Fixed dose ACT with shorter courses and fewer tablets may be key determinants to ease of administration and compliance.

**Methods:**

Children aged one year to 13 years presenting with uncomplicated *Plasmodium falciparum *malaria were recruited in Ibadan, south-western Nigeria. A total of 250 children each were randomly assigned to receive three doses of artesunate/sulphamethoxypyrazine/pyrimethamine (AS + SMP) (12 hourly doses over 24 hours) or three doses of artesunate/amodiaquine (AS + AQ) (daily doses over 48 hours). Efficacy and safety of the two drugs were assessed using a 28-day follow-up and the primary outcome was PCR- corrected parasitological cure rate and clinical response.

**Results:**

There were two (0.4%) early treatment failures, one in each treatment arm. The PCR corrected cure rates for day 28 was 97.9% in the AS + AQ arm and 95.6% in the AS + SMP arm (p = 0.15). The re-infection rate was 1.7% in the AS + AQ arm and 5.7% in the AS + SMP arm (p = 0.021). The fever clearance time was similar in the two treatment groups: 1 - 2 days for both AS + SMP and AS + AQ (p = 0.271). The parasite clearance time was also similar in the two treatment groups with 1 - 7 days for AS + SMP and 1 - 4 days for AS + AQ (p = 0.941). The proportion of children with gametocytes over the follow-up period was similar in both treatment groups. Serious Adverse Events were not reported in any of the patients and in all children, laboratory values (packed cell volume, liver enzymes, bilirubin) remained within normal levels during the follow-up period but the packed cell volume was significantly lower in the AS + SMP group.

**Conclusions:**

This study demonstrates that AS + SMP FDC given as three doses over 24 hours (12-hour intervals) has similar efficacy as AS + AQ FDC given as three doses over 48 hours (24-hour interval) for the treatment of uncomplicated *Plasmodium falciparum *malaria in children in Nigeria. Both drugs also proved to be safe. Therefore, AS + SMP could be an alternative to currently recommended first-line ACT with continuous resistance surveillance.

## Background

In Nigeria, nearly 110 million of clinical cases of malaria are diagnosed per year, translating into about 50% of the adult population experiencing at least one malaria episode per year, while young children can have up to two to four attacks of malaria annually [1]. In addition to the direct health impact of malaria on the Nigerian population, the economic loss linked to malaria in this country is estimated to ~132 billion Naira (±878 million USD) per year. In order to combat this devastating situation, the Nigerian authorities developed in 1996 a first National Malaria Control Policy, and in 1999 the Roll Back Malaria (RBM) programme was initiated.

National drug efficacy trials conducted in 2002 in Nigeria demonstrated that the first line treatments then employed, chloroquine and sulphadoxine/pyrimethamine (SP) were no longer adequate and in 2005, the highly efficacious artemisinin-based combination therapy (ACT) was introduced in the country [1]. Artemether-lumefantrine was elected first-line treatment for uncomplicated malaria with artesunate-amodiaquine (AS + AQ) recommended as an alternative [2]. Nowadays, SP is not recommended for the treatment of uncomplicated malaria due to its high resistance which can be as high as 35% but it is still in use for intermittent preventive therapy (IPT) in pregnant women. Similarity in the structure of SP and SMP denotes a potential for cross resistance [1,2].

Although the use of ACT for the treatment of uncomplicated malaria has been introduced several years ago, its utilization in the field is still below expected levels. The reasons explaining this situation include the poor availability and/or relatively high cost of ACT on the African market. One of the key elements of the RBM strategy is that malaria patients should have access to appropriate and adequate treatment within 24 hours of the onset of symptoms [1,2]. An anti-malarial drug to be used at home must, therefore, be safe, effective, affordable, easy to administer and preferably available in a single dose package. Following these lines, fixed-dose combinations (FDC) are preferred to co-blistered drugs as they prevent inadequate dosing (preventing drug resistance) and contribute to increase treatment compliance.

In view of the ideal profile to be found in an anti-malarial drug, the combination of artesunate and sulphamethoxypyrazine/pyrimethamine (AS + SMP) represents an interesting treatment option in case first-line drugs are not readily available. In fact, this product is easy to use, safe, and has previously demonstrated high efficacy in several endemic areas whether it is taken under a 24- or 48- hour regimen [3-5]. Previously offered as a co-blistered drug [6,7], the AS + SMP combination is now available as a FDC, which can easily be taken by malaria patients (three tablets only). In addition, since the SMP component has broad-spectrum anti-microbial activity, it also presents the interesting advantage of offering ancillary benefits against infections that may have been wrongly diagnosed as malaria [8,9].

In order to evaluate the safety and efficacy of the 24-hour therapy of AS + SMP FDC in south-west Nigeria, a cohort of children suffering from uncomplicated malaria were treated either with this ACT, or with a 48-hour therapy of AS + AQ FDC.

## Methods

### Study site

This study was conducted at the University College Hospital and at the Oni Memorial Children's Hospital both located in the urban areas of Ibadan, Oyo State, Nigeria. The intensity of malaria transmission varies considerably throughout Nigeria. Ibadan is located in the forest savannah woodland zone with average rainfall of 975 - 1474 mm/year [10]. Malaria, mainly caused by *Plasmodium falciparum*, is endemic in the region with a six-month transmission season (May-October) reaching its peak in August [11]. The overall entomological inoculation rates for *Anopheles gambiae s.l*. range from 18 to 145 infective bites per person per year [2]. Specifically in south-west Nigeria, the reported seasonal (six-month) entomological inoculation rates were 128.7 in 2001 and 131.3 in 2002 [11].

This study was approved by the Joint Ethics Committee of University of Ibadan and University College Hospital, Ibadan, Nigeria. The study was conducted in accordance with all requirements of the ICH Guidelines for Good Clinical Practice as well as the requirements of the Declaration of Helsinki. Clinical trial approval was obtained from the National Agency for Food and Drug Administration and Control in Nigeria (NAFDAC). Written informed consent was obtained from all parents or guardians of eligible children prior to enrolment.

### Patient screening and recruitment

Children presenting to each of the two study sites were screened for eligibility and invited to participate in the study if they met the following inclusion criteria: aged between 1 and 13 years; body weight between 6 and 40 kg; history of fever in the previous 24 hours or measured fever (axillary temperature >37.5°C); mono-infection with *P. falciparum*, with parasitaemia in the range of 2,000-200,000 asexual parasites per microlitre of blood; and no general danger signs or signs of severe and complicated *falciparum *malaria as per WHO guidelines [12].

### Study design, randomization and treatment

A randomized, controlled, open-label trial design was used. Assuming a proportion of 94% patients cured (with PCR-corrected cure rate at day 28 as primary endpoint) with AS + SMP, an equal proportion of patients cured with AS + AQ, a sample of 250 patients (taking into account 10-15% loss to follow-up) was required in each treatment arm to detect the 6% difference in parasitological cure rates with a power of 80% (α = 0.025 one-sided). Sample size calculations were done using nQuery Advisor 5.0. The randomization code was computer-generated with stratification per treatment centre, from which treatment groups were assigned.

At enrolment, a physical examination was performed, weight and axillary temperatures recorded and a medical history was obtained from parents/guardians including presenting symptoms and current medication. A finger prick blood sample was obtained for thin and thick blood smears and blotted on filter paper for parasite genotyping. A blood sample was also obtained for assessment of haematological and biochemical parameters.

Eligible children were then assigned into one of the two treatment groups according to the randomization code. The treatments were given in the clinic by the recruiting physician. One group received three doses of artesunate/amodiaquine 100/206.2 mg tablets (Amonate^®^, Dafra Pharma ltd., Kenya), crushed mixed with clean water and administered at 0, 24 and 48 hours. The second group received artesunate/sulphamethoxypyrazine/pyrimethamine 100/250/12.5 mg tablets (Co-Arinate FDC^® ^Junior, Dafra Pharma Ltd., Kenya), crushed, mixed with water and administered at 0, 12 and 24 hours. Treatment doses were given based on the age of the patient: children under 7 years received 1/2 tablet per dose (50/153.1 mg As/AQ and 50/125/6.25 mg As/SM/P) while children aged 7 years and older received a full tablet per dose. They were then observed for 1 hour after drug administration for vomiting. If vomiting occurred within 30 min after administration, the full treatment dose was re-administered. If vomiting occurred between 30-60 min after administration, half the treatment dose was re-administered.

No prior therapy was given and the only concomitant therapy administered were antipyretic drugs in patients with temperatures ≥38.5°C.

### Follow-up

The children on 12 hourly regimen whose second dose fell at night were admitted for the treatment to be given by the nurses on duty to ensure treatment compliance. Clinical and parasitological evaluation (thin and thick smears) was done on each day of follow-up (days 1, 2, 3, 7, 14, 21 and 28), or on any other day if the child was feeling sick. Patients were called back on these days or visited at home. During each visit, a brief clinical history was obtained to assess new complaints and possible side effects and a physical examination was performed. The haemogram and biochemical analysis (bilirubin, creatinine and liver enzymes AST, ALT) were done on days 0, 7 and 14 to detect possible significant abnormalities. Filter paper blood samples for parasite genotyping were obtained on day 28 or earlier if presented with repeat of the symptoms. Patients were excluded from the study if they withdrew consent, left the study area or reported taking anti-malarial medication during the follow-up period.

Response to drug treatment was assessed using modified WHO clinical classification system: all patients were not febrile at presentation, hence a temperature < 37.5°C was not an exclusion criterion and patients were followed up for 28 days in this area of intense transmission. The clinical classification system consisted of adequate clinical and parasitological response (ACPR), late parasitological failure (LPF), late clinical failure (LCF) and early treatment failure (ETF). The cure rates on day 28 were adjusted on the basis of the PCR genotyping results of paired samples for patients with recurrent parasitaemia after day 14 of starting treatment.

### Laboratory evaluation

A finger prick blood sample was taken to prepare thick and thin blood smears on days 0, 1, 2, 3, 7, 14, 21, 28 and on any other (unscheduled) visit. The slides were air dried, stained with 10% Giemsa for 20 min and read independently by two technicians. Parasite density (the number of parasites and gametocytes per μl) was calculated by counting parasites against 200 leukocytes and assuming a leukocyte count of 6,000/μl of blood. A slide was considered negative after reading at least 200 power fields. Thin blood smears were specifically used to identify *Plasmodium *parasite species. All qualitative discordant slides and slides with a parasite density difference ≥50% were read by a third microscopist. For quality control 10% of randomly selected slides were reread by an independent microscopist, not involved in the study.

Filter paper blood spots were collected on days 0, 28 or any other day of recurrent parasitaemia. Genotypes of the parasite population in each sample collected from patients with microscopically confirmed *P. falciparum *infections at enrollment, and during follow-up if the patients were parasitaemic on or before D28 were determined using PCR techniques. Analysis of genetic polymorphisms was performed on paired primary and post-treatment parasites samples obtained from the two treatment groups. Paired primary and post-treatment parasites were analysed using parasite loci that exhibit repeated numbers of polymorphisms to distinguish true treatment failures from new infections. Briefly, block 2 of *MSP-1 *(merozoite surface proteins-1), and the Block 3 of *MSP-2 *(merozoite surface protein-2) and region II of *GLURP *were amplified by two rounds of PCR using primers and amplification conditions [12,13]. Ten microliters of the PCR products were resolved by electrophoresis on a 2% agarose gel and sized against a 100-base pair molecular weight marker (New England Biolabs, Beverly, MA). The banding pattern of the post-treatment parasites were compared with matched primary parasites. Post-treatment and primary infection parasites showing identical bands were considered as true treatment failure, while non-identity will indicate a new infection [13,14]. Patients failing treatment within 28 days were retreated with oral quinine and were regarded as recrudescence or re-infection.

### Statistical analysis

The case report forms were double checked to ensure complete and accurate data collection. Dual data entry was done by two clerks after which data was compared, corrected and validated by the data manager. Data analysis was done using Epi Info version 6 and SPSS version 11. The primary analysis was a non-inferiority analysis (with clinically relevant difference of 6% and one-sided significance level of α = 0.025) based on PCR-corrected adequate clinical and parasitological response (ACPR) on day 28 as primary efficacy endpoint [15]. Secondary endpoints included gametocyte carriage, fever and parasite clearance time and packed cell volume levels. Chi-square and Fisher exact or Yate's correction tests were used to compare proportions between treatment groups. Normally distributed continuous variables were compared using Student's t test between two independent groups while non-parametric tests (Wilcoxon rank-sum) were used to analyse skewed data. Kaplan Meier was used to analyse the significance between the rates of re-infection in the two treatment arms. Two tailed p-values <0.05 were considered statistically significant. The Intention-To-Treat (ITT) population including all patients who were randomized to either of the two treatment groups and received at least one dose of study medication was used for safety analysis. The Per-Protocol population (PP) including all patients that received treatment and were not lost to follow-up was used for the efficacy analysis.

## Results

### Patients characteristics

**Figure 1:**
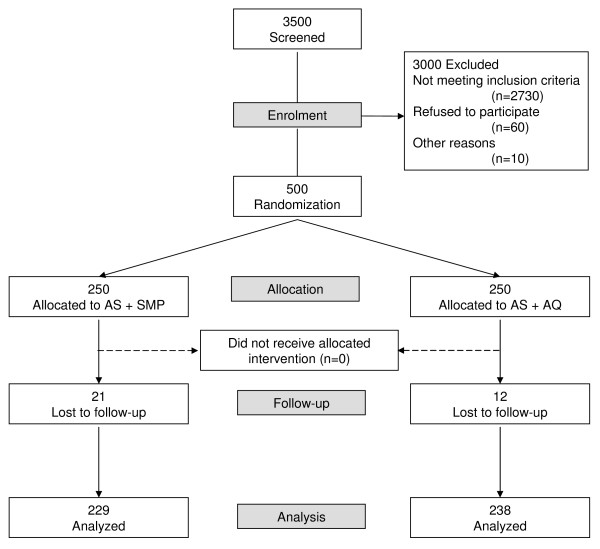
**Trial profile**.

Out of 3,500 children screened for malaria, 500 were enrolled into the trial; 250 were assigned to the AS + AQ treatment arm and 250 to the AS + SMP arm (Figure 1). Thirty three children (6.6%) were lost to follow-up, of which 21 in the AS + SMP arm and 12 in the AS + AQ arm. Both treatment groups were comparable in terms of baseline demographic, clinical, parasitological and laboratory characteristics (Table 1).

**Table 1:** Enrollment characteristics of children with uncomplicated malaria (ITT)

Characteristics	AS + SMP (n = 250)	AS + AQ (n = 250)	p-value
**Sex **(M:F)	128:122	136:114	0.299
			
**Age **(months)			
Mean ± SD	61.88 ± 38.139	60.77 ± 35.93	0.739
Range	12 - 156	12 - 156	
Proportion			
With age < 5 years	49.2%	58.8%	0.031
			
**Weight**			
Mean ± SD	17.51 ± 7.01	17.29 ±6.90	0.729
Range	6 - 40	7 - 40	
			
**Temperature **(Day 0)			
Mean ± SD	37.91 ± 1.15	37.83 ± 1.17	0.441
Range	35.5 - 40.5	35.3 - 40.7	
			
**Parasite Density **(Day 0)			
Median	15549.00	11673.50	0.078
Range	2009 - 199950	2020 - 199200	
			
PCV* (Day 0)			
Mean ± SD	29.79 ± 5.18	29.94 ± 5.09	0.748
Range	12 - 42	12 - 41	
			
AST* (Day 0)			
Mean ± SD	38.76 ± 14.66	38.88 ± 15.54	0.929
Range	10 - 95	10 - 101	
			
ALT* (Day 0)			
Mean ± SD	31.10 ± 16.63	30.27 ± 15.29	0.559
Range	10 - 99	10 - 91	
			
CRT* (Day 0)			
Mean ± SD	0.56 ± 0.26	0.56 ± 0.24	0.860
Range	0.20 - 1.30	0.20 - 1.20	
			
**Total Bilirubin **(Day 0)			
Mean ± SD	0.58 ± 0.28	0.62 ± 0.27	0.191
Range	0.10 - 1.70	0.10 - 1.30	
			
**WBC **(Day 0)			
Median	7450.00	7500.00	0.735
Range	1200 - 38000	1300 - 24600	

PCV = packed cell volume; AST = aspartate aminotransferase; ALT = alanine aminotransferase; CRT = creatinine

### Parasitological cure rates

There were two early treatment failures, one in each treatment arm (0.4%). The PCR corrected cure rates for day 28 was 97.9% in the AS + AQ arm and 95.6% in the AS + SMP arm (p = 0.15). The re-infection rate was 1.7% in the AS + AQ arm and 5.7% in the AS + SMP arm (Table 2, 3 and Additional file 1) (p = 0.021).

**Table 2:** Therapeutic response (PP)

Characteristics	AS + SMP (n = 229)	AS + AQ (n = 238)	p-value
**Fever clearance time (Days)**			
**Median (Range)**	1.00 (1 - 2)	1.00 (1 - 2)	0.271
			
**Parasite clearance time (Days)**			
**Median (Range)**	1.00 (1 - 7)	1.00 (1 - 3)	0.941
			
**Proportion with Fever**			
Day 1	32 (12.9%)	16 (6.5%)	0.016
Day 2	5 (2.0%)	5 (2.0%)	-
Day 3	3 (1.2%)	3 (1.2%)	-
**Proportion with Parasite**			
Day 1	66 (28.8%)	70 (29.4%)	0.888
Day 2	12 (5.3%)	9 (3.8%)	0.441
Day 3	3 (1.3%)	0 (0.0%)	0.117^+ ^
**Proportion with Gametocyte**			
Day 0	16 (7.0%)	16 (6.7%)	0.910
Day 1	18 (7.9%)	15 (6.3%)	0.511
Day 2	17 (7.4%)	18 (7.6%)	0.954
Day 3	17 (7.4%)	13 (5.5%)	0.387
Day 7	13 (5.7%)	11 (4.7%)	0.636
Day 14	8 (3.5%)	5 (2.2%)	0.390
Day 21	2 (0.9%)	3 (1.3%)	0.660
Day 28	5 (2.2%)	8 (3.5%)	0.408
**Treatment outcome**			
ACPR*	219 (95.6%)	233 (97.9%)	0.151
Recrudescence	9 (3.9%)	4 (1.7%)	0.135
Re-infection++	13 (5.7%)	4 (1.7%)	0.021
ETF*	1 (0.4%)	1 (0.4%)	-

Fisher's **^+^**

*ACPR = adequate parasitological and clinical response; ETF = early treatment failure

++ Kaplan Meier

**Table 3:** Survival Characteristics of re-infection in the treatment groups

Group	Number of cases	Number of events	Number censored	Mean survival time in days (95% C.I)
AS/SMP	229	13	216(94.3%)	27.541 (27.249-27.834)
AS/AQ	238	4	234(98.3%)	27.882 (27.695-28.070)

### Fever, parasite and gametocyte clearance and anaemia

**Figure 2:**
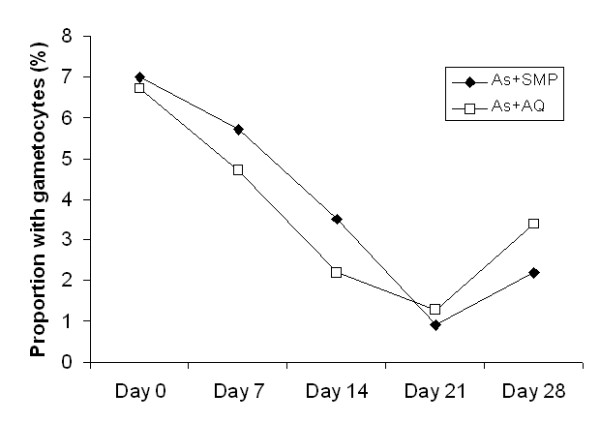
**Proportion of children with gametocytes up to 28 days after treatment with AS + SMP or AS +AQ**. The number of gametocytes per μl was calculated by counting parasites against 200 leukocytes (thick blood smear) and assuming a leukocyte count of 6,000/μl of blood.

**Figure 3:**
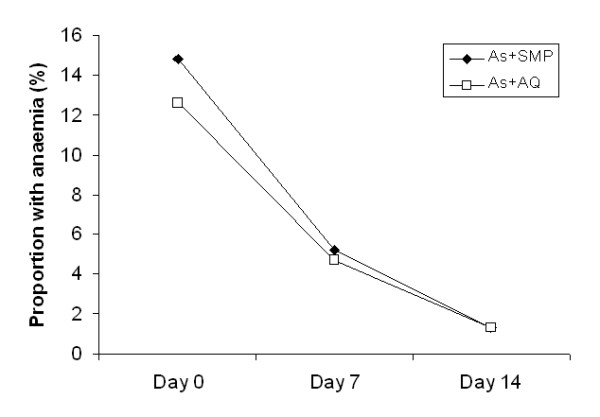
**Proportion of children with anaemia up to 14 days after treatment with AS + SMP or AS + AQ**.

The median fever clearance time was similar in the two treatment groups: 1 day for both AS + SMP and AS + AQ (p = 0.271). The median parasite clearance time was also similar in the two treatment groups with a range of 1 - 7 days for AS + SMP and 1 - 3 days for AS + AQ (p = 0.941). The proportions of children with gametocytes over the follow-up period were similar in both treatment groups. Sixteen children in each treatment arm had gametocytes on day 0 (7.0% for AS + SMP and 6.7% for AS + AQ) (Table 2). On day 28, the proportion of children with gametocytes was reduced to 2.2% for AS + SMP and 3.4% for AS + AQ (p = 0.408) (Figure 2). On day 14, the proportion of children with anaemia was reduced to 1.3% both in AS + SMP and AS + AQ treatment group (Figure 3).

### Adverse events

Serious adverse events were not reported in any of the patients. There was no significant difference in the proportions of patients reporting adverse events in the two treatment groups. Five patients (2.2%) treated with AS + AQ reported one or more of the following adverse events: vomiting (n = 1), excessive sleepiness (n = 2), abdominal pain (n = 1), and weakness (n = 3). Three patients (1.3%) treated with AS + SMP reported vomiting, one of these patients also reported nausea as adverse event. None of these patients needed to be admitted as a result of these events. For all children, laboratory values (packed cell volume, liver enzymes, bilirubin) remained within normal levels during the follow-up period (Table 4).

**Table 4:** Haematological and biochemical parameters (ITT)

	Drug type	Day 0	Day 7	Day 14
**Total bilirubin**				

Mean ± SD	AS + SMP	0.58 ± 0.28	0.58 ± 0.28	0.53 ± 0.27
	AS + AQ	0.62 ± 0.27	0.59 ± 0.26	0.53 ± 0.27
p-value		0.191	0.718	0.982

PCV*				

Mean ± SD	AS + SMP	29.79 ± 5.18	30.69 ± 4.50	33.65 ± 4.07
	AS + AQ	29.94 ± 5.09	31.78 ± 4.39	34.96 ± 4.32
p-value		0.748	0.008	0.001

AST*				

Mean ± SD	AS + SMP	38.76 ± 14.66	37.79 ± 15.59	36.90 ± 13.71
	AS + AQ	38.88 ± 15.54	36.82 ± 15.26	36.41 ± 13.85
p-value		0.929	0.492	0.700

ALT*				

Mean ± SD	AS + SMP	31.10 ± 16.63	31.27 ± 15.50	29.93 ± 15.38
	AS + AQ	30.27 ± 15.29	30.04 ± 14.43	29.09 ± 14.68
p-value		0.559	0.365	0.545

CRT*				

Mean ± SD	AS + SMP	0.56 ± 0.26	0.55 ± 0.28	0.54 ± 0.27
	AS + AQ	0.55 ± 0.24	0.55 ± 0.25	0.53 ± 0.24
p-value		0.860	0.810	0.745

• PCV = packed cell volume; AST = aspartate aminotransferase; ALT = alanine aminotransferase;

• CRT = creatinine

## Discussion

This study is documenting the efficacy and tolerability of AS + SMP compared to the recommended artesunate-amodiaquine (AS + AQ) ACT for the treatment of uncomplicated *P. falciparum *malaria in children in an endemic area of south-western Nigeria. The study was based on a 28-day follow-up period.

Many African countries have adopted artemisinin based combination therapies (ACTs) as first-line therapy against uncomplicated malaria, particularly artemether-lumefantrine (AL) and AS + AQ. However based on field experience, there is a need to assess other forms of ACT in order to identify a suitable alternative to recommended treatments in case those are not readily available to the patients who urgently need to be treated. The 28-day cure rates observed in this study were 95.6% in the AS + SMP treatment arm compared to 97.9% in the AS + AQ treatment arm (p = 0.151) suggesting that the therapeutic efficacy of both drugs were similar. These findings are in agreement with previously reported studies on AS + SMP from other endemic and non-endemic malaria areas [3-7]. The observed AS + AQ cure rate is also similar to those stated in other studies [16-18]. The significantly higher under 5 year old children in the AS + AQ group would have possibly led to a lower inability to clear malarial parasites in the AS + AQ group as a result of their relatively low immunity. The loss to follow up rate was also significantly higher in the AS + SMP group than the AS + AQ group (21 versus 12) this may not be related to the drug efficacy as these losses were mainly due to movement to and from schools as well as other family social issues. The fact that 3,500 children had to be screened in order to attain the postulated sample size of 250 children per treatment arm suggests that a high proportion of the cases of fever in children are not due to malaria infection, but e.g. due to bacterial infections [9]. On the field, in the absence of parasitological confirmation, the anti-microbial profile of the SMP component of AS + SMP would have provided clinical benefits to such children even if malaria would have been wrongly diagnosed.

Gametocyte carriage before and following treatment were similar in the two treatment arms, which is expected to be the result of the action of artesunate [19]. Interestingly, sulphadoxine/pyrimethamine (SP) was shown to increase gametocyte carriage following treatment of uncomplicated malaria infection [20], but this does not seem to be the case for SMP. In fact, Sowunmi *et al *reported that, despite slower clearance of sexual parasitaemia in children treated with AQ + SMP compared with those treated with AL, treatment with the former was not associated with increased gametocyte carriage [19].

Both AS + SMP and AS + AQ treatments were well tolerated and the frequency of reported adverse events was similar in the two treatment arms. In reality, the reported adverse events were difficult to distinguish from signs and symptoms of malaria. It is notable that the reported frequency of those with itching treated with AS + AQ was low, similar to what was previously reported by Sowunmi *et al *in 1989 [21]. Serious adverse events such as icterus or intravascular haemolysis were not reported by any of the children. However the packed cell volume on follow up was found to be significantly lower in patients treated with AS + SMP and this may be related to high frequency (23.9% in males and 4.6% in females) of glucose six phosphate dehydrogenase (G6PD) deficiency in the study area [22]. This would have been substantiated if all patients were screened for G6PD Deficiency.

In summary, this study demonstrates that AS + SMP FDC given as three doses over 24 hours (12-hour intervals) has similar efficacy as AS + AQ FDC given as three doses over 48 hours (24-hour interval) in terms of fever and parasite clearance for the treatment of uncomplicated *Plasmodium falciparum *malaria in children in Nigeria. Both drugs also proved to be safe though AS + SMP has a higher re-infection rate. AS + SMP represents a good alternative to recommended first line treatments in areas where these are not available, or only offered at high costs. A continuous surveillance on the development of drug resistance is however essential as a result of potential cross resistance between SP and SMP.

## Competing interests

FHJ is an employee of the drug company Dafra Pharma. Dafra Pharma supported the study. Dafra Pharma developed and supplied the fixed dose medication artesunate/sulfamethoxypyrazine/pyrimethamine. The authors and the sponsor were involved in study design, trial monitoring and interpretation of the date and writing of the report. No honoraria were paid for running the trial to any of the co-authors.

## Authors' contributions

IAA contributed to the design of the study, project implementation (Principal Investigator) and wrote the manuscript. AGF and AS contributed to the design of the study, the performance of the research, and participated in the writing of the manuscript. FHJ contributed in the design of the study and the writing of the manuscript. All authors have read and approved the final manuscript.

## Supplementary Material

Additional file 1 **Kaplan Meier curve for rates of re-infection in the treatment arms AS+SMP and AS+AQ**. Click here for file
