# Supplementary figures and images for: An open randomized clinical trial in comparing two artesunate-based combination treatments on Plasmodium falciparum malaria in Nigerian children: artesunate/sulphamethoxypyrazine/pyrimethamine (fixed dose over 24 hours) versus artesunate/amodiaquine (fixed dose over 48 hours)
Source: Malar J. 2010 Dec 31;9:378. doi: 10.1186/1475-2875-9-378 (PMC3024282; doi:10.1186/1475-2875-9-378)

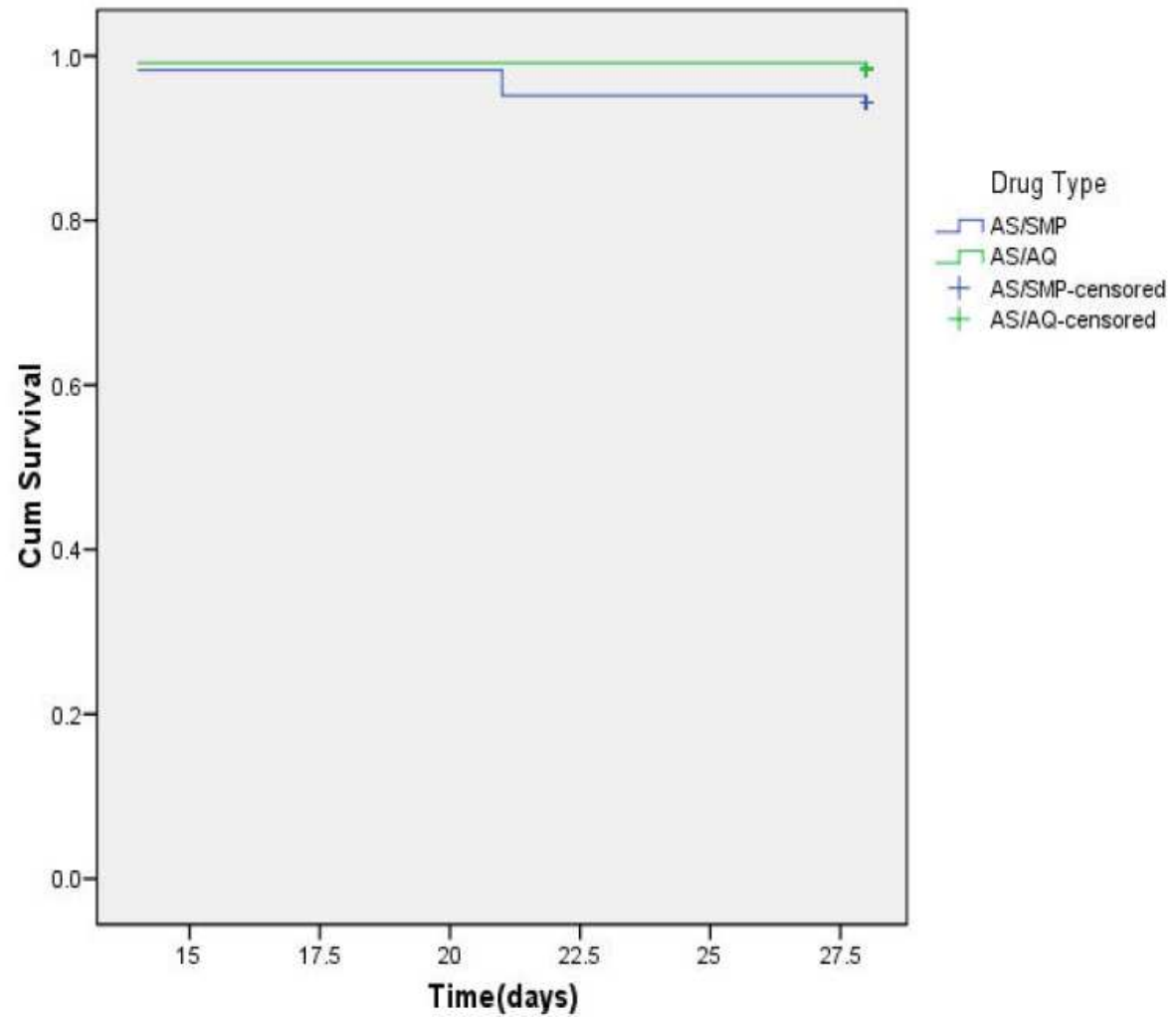

Supplement: Additional file 1 — Kaplan Meier curve for rates of re-infection in the treatment arms AS+SMP and AS+AQ. [file 1475-2875-9-378-S1.PDF]
